# Insights Into Antimicrobial Resistance From Dental Students in the Asia–Pacific Region

**DOI:** 10.1016/j.identj.2024.09.016

**Published:** 2024-10-05

**Authors:** Saba Ghafoor, Gabriela Salvadori, Shiho Kino, Vy Thi Nhat Nguyen, Tam Thi-Thanh Nguyen, Miho Ishimaru, Antônio Pedro Ricomini-Filho, Cassiano Kuchenbecker Rösing, Dileep De Silva, Jun Aida, Belinda Farias Nicolau, Ratilal Lalloo, Roger Junges

**Affiliations:** a Institute of Oral Biology, Faculty of Dentistry, University of Oslo, Oslo, Norway; b Department of Oral Health Promotion, Graduate School of Medical and Dental Sciences, Tokyo Medical and Dental University, Tokyo, Japan; c Faculty of Odonto-Stomatology, Hue University of Medicine and Pharmacy, Hue University, Hue, Vietnam; d Faculty of Odonto-Stomatology, The University of Medicine and Pharmacy at Ho Chi Minh City, Ho Chi Minh City, Vietnam; e The Institute of Education, Tokyo Medical and Dental University, Tokyo, Japan; f Piracicaba Dental School, University of Campinas, Piracicaba, Brazil; g Faculty of Dentistry, Federal University of Rio Grande do Sul, Porto Alegre, Brazil; h Faculty of Dental Sciences, University of Peradeniya, Peradeniya, Sri Lanka; i Faculty of Dental Medicine and Oral Health Sciences, McGill University, Montreal, Québec, Canada; j The University of Queensland, School of Dentistry, Brisbane, Australia

**Keywords:** Dental education, One Health, Antimicrobial resistance, Microbiology, Antimicrobial stewardship, Drug prescriptions

## Abstract

**Background:**

Dentists, as prominent prescribers, are key stakeholders in addressing the antimicrobial resistance (AMR) crisis. Dental students’ perceptions about the topic have been underexplored in the Asia–Pacific region, a key location for the development and spread of AMR. Thus, the aim of this study was to evaluate the awareness and confidence to prescribe antimicrobials amongst dental students studying in the region.

**Methods:**

Students from 15 dental schools in 4 countries were invited to participate in a cross-sectional online survey during 2022–2023. A previously validated and standardised 14-item instrument was utilised.

**Results:**

In all, 1413 responses were collected from Australia (n = 165), Sri Lanka (n = 112), Japan (n = 173), and Vietnam (n = 963). Of those, 201 were from final-year students (14.2%). On a scale from 1 to 10, awareness on AMR was placed at a mean (SEM) priority of 8.09 (0.05). With regards to target areas to address for mitigation of the AMR crisis, participants placed general public awareness at the top (mean [SEM] 8.53 [0.05]). Final-year students presented a mean (SEM) level of confidence to prescribe antibiotics of 6.01 (0.14) on a scale from 1 to 10, whilst 59.7% and 56.8% indicated feeling pressured to prescribe by patients or when lacking time, respectively. Final-year students participating in research activities assigned a higher priority to AMR compared to their peers not involved in research (mean [SEM] 8.6 [0.19] vs 7.81 [0.16]; *P* = .01).

**Conclusions:**

This study highlights a need for increased awareness and confidence to prescribe amongst dental students in the Asia–Pacific region, an understudied population thus far. To mitigate this issue, the implementation (followed by assessment) of local educational and antibiotic stewardship initiatives is warranted.

## Introduction

Antimicrobials are a central pillar of modern health care; however, their effectiveness is under threat due to the antimicrobial resistance (AMR) crisis. Globally in 2019, estimates indicate 4.95 million deaths associated with AMR, 1.27 million of which were directly attributed to AMR.^1^ High population numbers and densely populated cities with many cases of unplanned urbanisation create a particular concern for the emergence and spread of infectious diseases in the Asia–Pacific region.^2^, ^3^, ^4^ Further, a diverse mosaic of high-income and low-to-middle income countries (LMIC) with often difficult-to-access health care creates challenges for public health and potential hotspots for the emergence of AMR.^3^^,^^4^ To address causes and provide solutions sustainable in the long term, it is pivotal that close surveillance and collaboration across multiple sectors are aimed at optimising the health of humans, animals, and the environment as a whole. Such an integrated approach, engaging varying levels of society to work together, is defined as One Health and is particularly important in the global response to the AMR crisis.^5^

The Global Action Plan on AMR^6^ places the optimisation of antibiotic use at its core, recognising that the misuse of these drugs is one of the main drivers of AMR. Antibiotic use has been increasing globally over recent decades^7^^,^^8^; however, divergent patterns can be observed across countries partially due to different economic developmental stages and local policies.^8^^,^^9^ In fact, the growth in wealth expected for many LMIC in the Asia–Pacific region will likely generate a greater demand for the use of antibiotics in both humans and animals.^9^, ^10^, ^11^ In addition, distinct country health profiles and different levels of development of health care education could play a key role in the implementation of contemporary evidence-based practices. The majority of antibiotics used in humans are prescribed to outpatients.^12^ Despite the scarcity of data for a variety of locations, including in the Asia–Pacific region, evidence indicates that dentists are significant contributors to this group, prescribing about 10% of outpatient doses.^13^ Further, concerns have been raised regarding the overall appropriateness of antibiotic prescriptions used in dental procedures.^14^, ^15^, ^16^, ^17^

In order to optimise the use of antibiotics, stewardship programmes^18^, ^19^, ^20^ and other intervention strategies^21^^,^^22^ need to be implemented locally both in practice and in dental curricula. Recently, the FDI World Dental Federation has made significant efforts in communicating the importance of antimicrobial stewardship practices in dentistry.^13^ The establishment of frameworks for antimicrobial stewardship has been proposed both in dental education^23^ and in practice.^20^^,^^24^ However, there is a lack of standardisation and, often, teaching of AMR is diluted in microbiology and pharmacology and in each specific dental specialty during clinical training.^25^^,^^26^ In addition, the social context for students is likely to influence their perceptions on the AMR crisis and may further impact prescription practices.^27^^,^^28^

Recent multicentre studies monitoring knowledge and practices on AMR in dentistry have varied in size and mostly included participants from Europe and the Americas^29^, ^30^, ^31^, ^32^; thus, it is important to assess the perceptions of students from the Asia–Pacific region regarding the AMR crisis. The increase in knowledge generated by monitoring perceptions, awareness, and practices in both patients and prescribers is instrumental for the development of targeted interventions, as it can illuminate gaps in knowledge and highlight specific local challenges.^33^^,^^34^ In addition, the next generation of prescribers are key stakeholders in the fight against AMR not only in terms of directly reducing the number of antibiotic prescriptions but also in terms of engaging and advocating through their local communities. This is particularly important in regions that lack regulation and where antibiotics can be obtained without prescription, as is the case in a variety of countries in the Asia–Pacific region.^33^^,^^35^ Thus, the benefits of enhancing prescriber awareness on AMR are likely to transcend the clinical setting and can potentially lead to reductions in antibiotic usage in society generally.

Here, we present, to the best of our knowledge, the first large multicentre study focussed on AMR perceptions amongst dental students from the Asia–Pacific region, a largely underexplored population. We utilised an instrument recently developed and employed by our group^29^ that is tailored to dental students and allows for standardisation in comparative studies. A panel of 4 countries were included in the survey: Australia (4- and 5-year programmes), Sri Lanka (5-year programme), Vietnam (6-year programme), and Japan (6-year programme with a greater focus on dentistry in the last 4 years). Whilst there are variations in the curricular structure and likely in the format of teaching in each location and institution, the basic science behind AMR and stewardship are taught in each of the countries together with microbiology and pharmacology in the initial years. The content is further revisited in the clinical teaching and rotations, with a particular focus on infection control and antibiotic prescription practices. Thus, the aims of this study were to assess awareness regarding AMR in all dental students and to evaluate confidence to prescribe and interest in further education in final-year students at the surveyed institutions. By unraveling perceptions regarding AMR and confidence to prescribe antibiotics, the objective was to provide insights that may contribute to the development of intervention strategies and educational frameworks within dental and health care sciences in the Asia–Pacific region.

## Methods

### Study participants

Dental students from 15 universities located in Australia (n = 7), Sri Lanka (n = 1), Japan (n = 3), and Vietnam (n = 4) participated in this voluntary and anonymous survey. A census-style survey was employed; thus, attempts were made to reach out to all students at the participating institutions. No compensation was offered, and data collection took place between July 2022 and July 2023.

### Survey content and implementation

The questionnaire employed in the survey was previously developed by our group^29^ and contained only minor adaptations in content to ensure standardisation. The instrument collects data on perception and awareness of AMR amongst all dental students enrolled in each university. Further, final-year students were invited to answer 2 additional sections focussed on confidence and challenges to prescribe antibiotics generally and in specific clinical scenarios as well as interest in further education by presenting a variety of topics pertinent to AMR. The survey was hosted online and distributed to students via email, and up to 3 reminders to complete the survey were sent. Estimated total time for completion of the questions was 5 to 10 minutes, as the questionnaire was designed to contain primarily close-ended questions to avoid fatigue and maximise compliance. To ensure accuracy, translation to Japanese/Vietnamese and back-translation to English were performed by native speakers.

### Data analysis

Data were extracted from the online server, translated into English when necessary, and imported into Microsoft Excel and IBM SPSS 29.0 for analysis. In addition to data visualisation, descriptive statistics were also performed using GraphPad Prism 9. Means were reported together with the standard error of the mean (SEM). For nonparametric data, discrete data were compared with Kruskal–Wallis followed by Dunn's post hoc test and numerical means were compared with Mann–Whitney test. For parametric data, numerical comparisons were performed with Student *t* test. Significance level was set at 0.05.

### Ethical aspects

This study complied with the Declaration of Helsinki and the standards provided by each of the countries involved in the project. Ethical approval was obtained prior to the commencement of the project and approved as follows: Australia (2022/HE000412), Sri Lanka (ERC/FDS/UOP/2022/11), and Japan (C2022-013). Ethical approval for this study was waived by the Institutional Ethics Committee of Hue University of Medicine and Pharmacy in Vietnam because of its anonymous design and non–health-related nature of the survey. In the link provided to students, the cover letter contained information about the project and consent was collected prior to the presentation of the questions.

## Results

### Study sample and characteristics

A total of 1413 responses were collected from 15 dental schools in Australia, Sri Lanka, Japan, and Vietnam. Most of the participants identified as female (60.4%), and mean age (SEM) was 22.36 (0.09; median, 22). Involvement with research activities ranged from 12.6% (Vietnam) to 33.9% (Sri Lanka). Final-year students represented 201 of the responses (14.2%). Demographic data for the study sample of each country are available in Table 1. Calculation of the response rate is challenging, as determining the exact number of students who received or opened the survey was not possible. However, based on the total number of students enrolled in each institution, response rates of 42% in Vietnam, around 27% each in Japan and Sri Lanka, and less than 10% in Australia are estimated, resulting in an average response rate of 26%. Number of responses obtained by year in each country are available in Table S1.

**Table 1:** Characteristics of the participants from the Asia–Pacific region that participated in the survey during 2022–2023.

	Population	Australia	Sri Lanka	Japan	Vietnam
Participants, No. (%)	1413 (100)	165 (100)	112 (100)	173 (100)	963 (100)
Age, mean (SEM) median, y	22.36 (0.09) 22	23.55 (0.42) 22	24.61 (0.15) 25	24.39 (0.29) 23	21.52 (0.09) 21
Sex, No. (%)					
Female	854 (60.4)	109 (66.1)	68 (60.7)	92 (53.2)	585 (60.7)
Male	540 (38.2)	55 (33.3)	43 (38.4)	77 (44.5)	365 (37.9)
Nonbinary	17 (1.2)	1 (0.6)	0 (0)	4 (2.3)	12 (1.2)
Prefer to self-describe	2 (0.1)	0 (0)	1 (0.9)	0 (0)	1 (0.1)
Research activities, No. (%)	226 (16)	38 (23)	38 (33.9)	29 (16.8)	121 (12.6)
Final-year students, No. (%)	201 (14.2)	35 (21.2)	14 (12.5)	39 (22.5)	113 (11.7)
Interest in work placement for final-year students, No. (%)					
Private-sector	152 (75.6)	31 (88.6)	5 (35.7)	20 (51.3)	96 (85)
Public-sector	76 (37.8)	19 (54.3)	11 (78.6)	12 (30.8)	34 (30.1)
Academia	31 (15.4)	5 (14.3)	5 (35.7)	12 (30.8)	9 (8)
Other	9 (4.5)	1 (2.9)	0 (0)	4 (10.3)	4 (3.5)
Unsure	15 (7.5)	3 (8.6)	1 (7.1)	7 (17.9)	4 (3.5)

### Dental students’ awareness on AMR

**Fig. 1:**
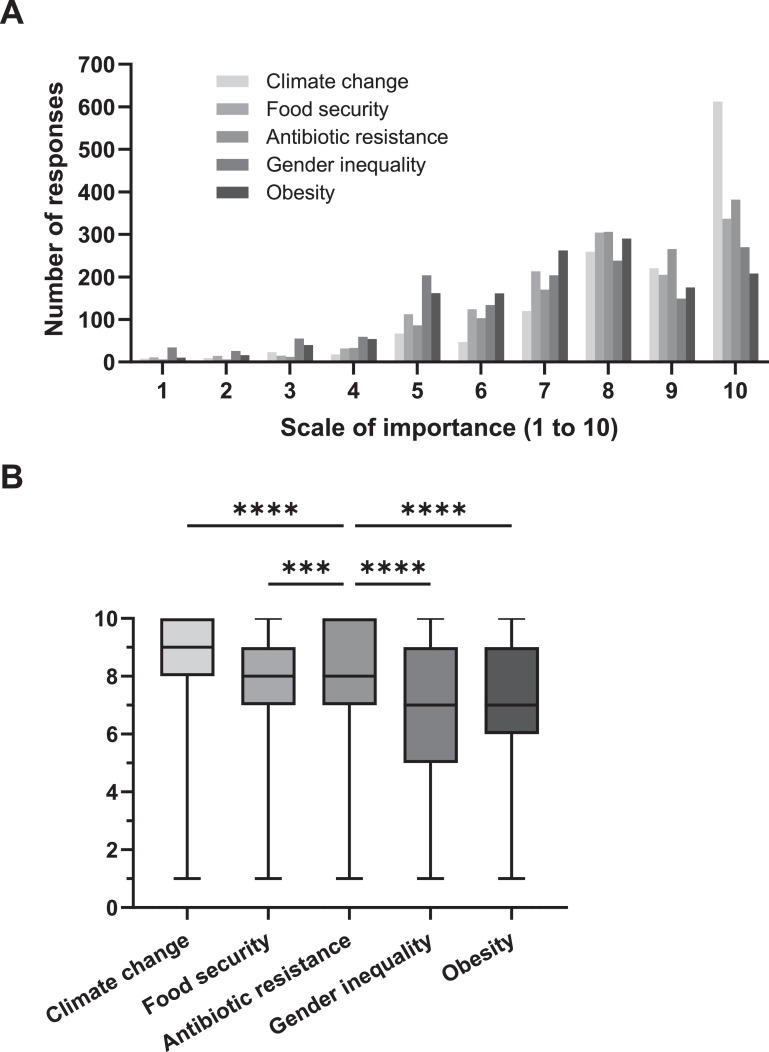
Perceived awareness from all dental students in the Asia–Pacific region regarding 5 proposed global challenges on a scale from 1 to 10. **A**, Histogram for number of responses regarding each of the challenges. **B**, Boxplot with lines indicating the median and whiskers indicating range. ***P < .001, ****P <  .0001.

For all dental students, on average, the 5 presented global challenges were considered important, as shown in the left-skewed distribution of the histograms (Figure 1A). The highest priority was climate change (mean [SEM], 8.53 [0.05]), followed by AMR (mean [SEM], 8.09 [0.05]) and food security (mean [SEM], 7.79 [0.05]). Direct comparison showed statistically significant differences between AMR and the other challenges (Figure 1B). When looking only at final-year students, the participants involved with research expressed greater AMR awareness compared with their peers who were not involved in research activities (mean [SEM], 8.6 [0.19] vs 7.81 [0.16]; *P* = .01). A similar trend of greater awareness was also observed with regards to the other 4 challenges, but these differences were not statistically significant.

**Fig 2:**
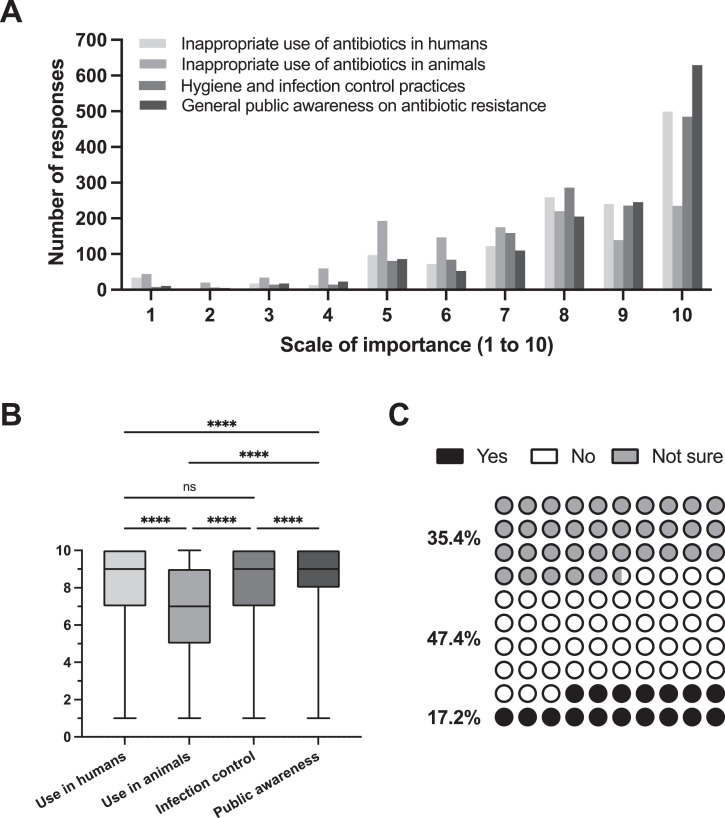
General areas that should be targeted to combat the antimicrobial resistance crisis according to all dental students in the Asia–Pacific region. **A**, Histograms of all responses on a scale from 1 (not at all important) to 10 (extremely important). **B**, Boxplot of the responses with lines indicating the median and whiskers indicating range. **C**, Participants’ familiarity with the concept of One Health. ****P < .0001.

With regards to which areas to target to mitigate AMR, addressing the inappropriate use of antibiotics in animals was perceived as the least important (mean [SEM], 6.99 [0.06]; Figure 2A and 2B) by dental students from all years. In addition, 146 respondents (10.3%) did not know how to position themselves regarding inappropriate use of antibiotics in animals, in comparison to 55 (3.9%) regarding antibiotic use in humans, 40 (2.8%) for hygiene practices, and 28 (2%) for public awareness. The highest priority was reported to be public awareness (mean [SEM], 8.53 [0.05]), followed by hygiene practices (mean [SEM], 8.3 [0.04]) and inappropriate use of antibiotics in humans (mean [SEM], 8.2 [0.05]). There were statistically significant differences across all comparisons with the exception of inappropriate use in humans vs hygiene practices (Figure 2B). Only 17.2% of the participants had heard about the concept of One Health (Figure 2C), and this portion was even lower when looking only at final-year students (10%, n = 20). Further, the majority (93.9%) selected AMR as an important topic for dentists.

### Final-year students’ confidence to prescribe antibiotics and interest in further education

**Fig. 3:**
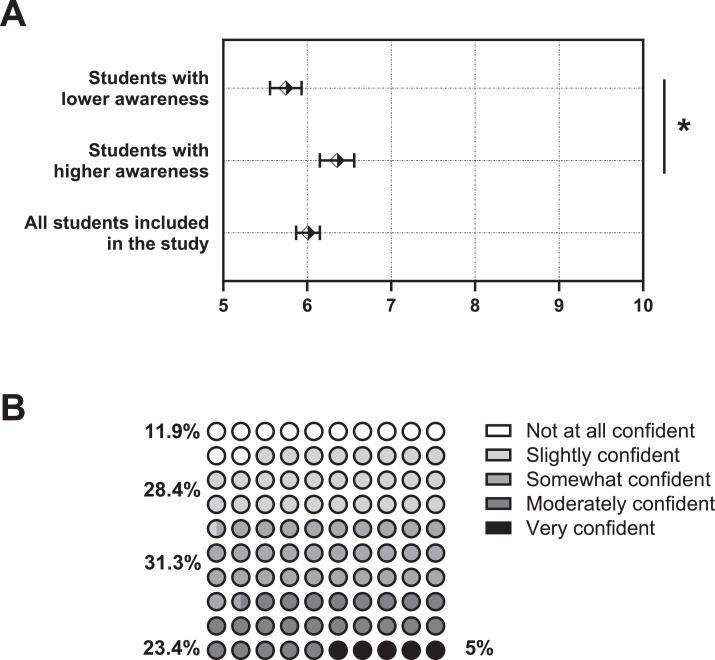
Confidence of final-year dental students to prescribe antibiotics. **A**, Confidence levels on a scale from 1 to 10. Symbols show averages, with error bars representing standard error of the mean (SEM). “Lower awareness” indicates students who marked the challenge of antimicrobial resistance (AMR) as 8 or below (n = 106), and “higher awareness” indicates students who marked AMR as either 9 or 10 in terms of priority (n = 90). **B**, Level of confidence in relative values of final-year students to communicate to patients when antibiotics are not necessary. *P < .05.

**Fig. 4:**
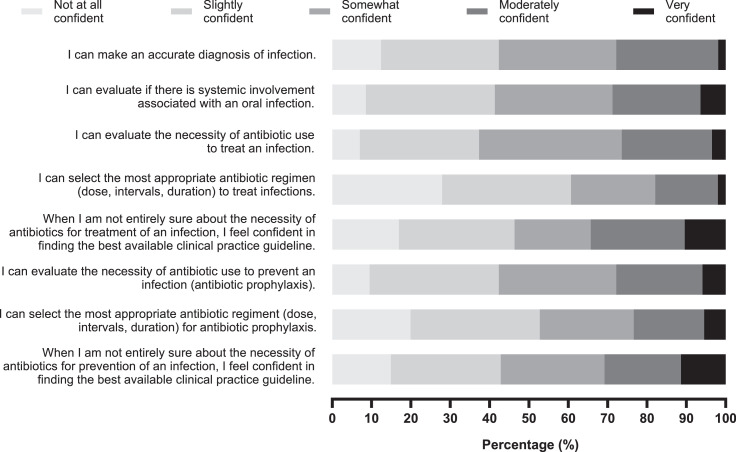
Level of confidence of final-year dental students in the Asia–Pacific region regarding antibiotic prescriptions for treatment (first 5 statements) and prevention of infections (last 3 statements). Relative values (%) of responses are represented on the x-axis.

Final-year students had a mean (SEM) level of confidence to prescribe of 6.01 [0.14] on a scale of 1 to 10 (Figure 3A). Participants who had marked AMR with a priority of 9 or 10 (Figure 1) expressed greater confidence to prescribe than those marking AMR with a priority of 1 to 8 (*P* < .05). The cutoff for awareness of AMR was selected due to data distribution. Only 5% of participants expressed being very confident to communicate with patients when antibiotics are not needed (Figure 3B). Participants strongly agreed (4.5%), agreed (21.9%), and somewhat agreed (33.3%) that they felt pressure to prescribe antibiotics when lacking time. Further, 5.5% strongly agreed, 24.9% agreed, and 26.4% somewhat agreed that they felt pressured by patients to prescribe. Only 1.5% and 10.4% selected to speak with patients about AMR very often and often, respectively. On a list of different clinical scenarios, participants were most confident to identify clinical guidelines for antibiotic prescription and least confident to select the most appropriate antibiotic regimen needed (Figure 4). For further education, the majority of final-year students expressed strong interest in all topics listed (Table 2). Small-group teaching and online courses were classified as very useful for 36.3% and 24.4% of the population, respectively.

**Table 2:** Interest of final-year students in receiving further education on selected topics with regards to antimicrobial resistance and prescription practices.

	Yes		No		Not sure	
	No.	%	No.	%	No.	%
Spread of resistance	179	89.1	16	8	6	3
Development of resistance	178	88.6	18	9	5	2.5
Drug interactions	184	91.5	11	5.5	6	3
Antibiotic prescription: treatment	187	93	11	5.5	3	1.5
Antibiotic prescription: prophylaxis	181	90	16	8	4	2
Antibiotic resistance in humans, animals, and the environment	167	83.1	24	11.9	10	5
Links between systemic diseases and oral conditions	185	92	12	6	4	2

### Country-specific data

Data specific to each country are available in Figures S1 to S4. The perceived awareness of the 5 global challenges varied across countries (panel A), whilst the pattern for the priority areas to focus on in order to combat AMR remained similar (panel B). Confidence to prescribe (panel C) was reported as being lowest in students participating from Japan (mean [SEM], 4.95 [0.36]), and the pattern of lower confidence to prescribe amongst students who ranked AMR as a lower priority is also observed in all countries. Further, students from all countries were interested in receiving more education about the topics listed. This finding was similar across countries, with the exception of Australia, where students showed less degree of interest. With regards to the One Health concept, familiarity was greater in Vietnam (19.8%) and Japan (17.3%) and lower in Sri Lanka (11.6%) and Australia (5.5%). Panels A and B show data for dental students in all years, whilst panels C through E show data for final-year dental students.

## Discussion

This study aimed to assess awareness regarding AMR amongst dental students from the Asia–Pacific region. Participants in this study identified AMR as a pressing global threat, together with climate change and food security, whilst obesity and gender inequality were ranked lower on the priority list. Further, general public awareness was identified as a central target area to slow the development of AMR. Regarding the aim to evaluate confidence in prescribing antibiotics (amongst final-year students), the average confidence to prescribe was rated a 6 on a scale from 1 to 10. Students who had rated AMR as a global priority as a 9 or 10 expressed higher confidence to prescribe. Almost 60% of final-year students reported feeling pressured to prescribe by patients or when lacking time. Less than 30% felt confident to communicate to patients when antibiotics were not needed. There was a high level of interest in receiving further education for the topics listed as expressed by final-year students.

The dental students from the Asia–Pacific region included in this study perceived the AMR threat as less of a priority when compared with dental students in Norway, Canada, and Brazil.^29^ On a scale from 1 to 10, students from the Asia–Pacific region rated the challenge of AMR as 8.09 compared with 8.44 in Norway, 8.59 in Canada, and 9.34 in Brazil. A parallel can also be drawn to a study in the United Kingdom in which dental students (n = 11) ranked AMR on average as a 9.^30^ Smaller studies with dental students in the Asia–Pacific region show concerning levels of knowledge in India^36^ and high levels of self-medication with antibiotics amongst dental students in Nepal^37^ and the United Arab Emirates.^38^ In Australia, a survey focussing on medical doctors, dentists, and veterinarians identified high levels of awareness regarding AMR across the groups; however, a significant degree of externalised responsibility was indicated by the participants.^39^ In Japan, a 2022 study reported potential for improvement in pharmacy students’ awareness and knowledge on AMR, having found levels similar to the general public in some areas.^40^

On a scale from 1 to 10, confidence to prescribe amongst students in the Asia–Pacific region was rated at 6.01, in comparison with 6.89 in Norway, 7.13 in Canada, and 8.22 in Brazil.^29^ Whilst Japanese students reported the lowest confidence to prescribe (4.95), the pattern of low confidence seemed consistent across countries in this survey. Findings from a recent US study using case vignettes identified an average confidence to prescribe of 3.3 out of 5, which would be equivalent to 6.6 out of 10, in third- and fourth-year dental students.^41^ In residents and faculty at the same institution, confidence was rated 7.62 and 7.84, respectively, on a scale from 1 to 10. Of note, confidence was assessed in relation to 7 specific case vignettes, not general confidence in clinical practice. Even though confidence to prescribe does not seem to be directly correlated with better knowledge or practices,^41^^,^^42^ efforts should be directed towards increasing confidence in dental students. In terms of further education, the majority of students were interested in receiving more information regarding all topics listed, spanning from basic to clinical sciences and public health. The demand was greater than in previous responses to this questionnaire, with the exception of Australia, which showed similar demand to previous research.^29^ Using different methodologies, other studies have also identified a high demand for more education regarding AMR as expressed by dental^31^ and medical students.^43^, ^44^, ^45^

Increasing public health awareness and adoption of good practices is central to mitigating the impact of AMR. Lack of knowledge regarding antibiotics and AMR drives the demand for antibiotics from the general public and lowers the chances of treatment compliance.^46^, ^47^, ^48^ In addition, the main source of information for people in the region has been reported to be their direct health care provider,^47^ which highlights the relevance of a well-equipped and engaged prescribing population and health care team. Whilst participants in this study identified the need for improvement in general public awareness as the top priority moving forwards, almost 60% reported to some extent feeling patient pressure to prescribe antibiotics. Further, less than 30% felt very confident or moderately confident in communicating with patients when antibiotics are not needed. In particular, data from Vietnam show that only 14.2% of final-year students felt very confident or moderately confident to communicate to patients when antibiotics are not needed. Further, use of antibiotics in animals was identified as less of a priority than the other areas suggested in the fight against AMR by participants in this study. This raises concerns, as antibiotics are routinely used in animal farming for treatment, prophylaxis, and growth promotion. Together with use in humans, use in animals constitutes one of the biggest drivers of AMR.^49^ Based on our data, a stronger focus on transdisciplinarity and integration of the One Health concept into dental curricula is warranted. Similar findings have been observed amongst undergraduate students at a university in Brunei, where only 25% of the students were aware of the use of antibiotics in agriculture and food-producing animals.^50^

The key strength of the study lies in the multicentre approach, including dental students from 4 diverse countries in the Asia–Pacific region, an underexplored area so far. Further, presentation of data separately for final-year students allows for the understanding of probable future scenarios, as these individuals are further along in their studies and will soon be in the field as professionals. Because raising awareness is key in the fight against AMR, one of our goals was also to create a communication channel and engage with local populations throughout the study. The creation of a standardised measurement instrument as a resource allowing for comparison across studies and in cohorts over time is likely to contribute to other research projects and educational activities in the field.

Limitations are related to varied levels of engagement from students in the different countries included, such as a higher number of responses collected from Vietnam as opposed to other countries, which can skew the descriptive analyses. In addition, caution should be exercised when interpreting survey studies because of the risk of nonresponse bias.^51^^,^^52^ Calculation of the response rate is challenging because it is difficult to determine the exact number of students who received or opened the survey. However, the high number of responses obtained is positive, and it is unclear whether a higher number of responses would influence the patterns observed in the results. In addition, the estimated average of 25% to 30% response rates is in line with previous studies employing similar strategies for data collection.^29^^,^^43^^,^^53^^,^^54^

Educational interventions within dentistry to clarify the appropriateness of antibiotic prescription may focus on the establishment of stewardship programmes focussed on monitoring rates and appropriateness of prescription, clinical response and adverse outcomes, as well as other patient-reported measures.^24^ The addition of elements of basic microbiology and strategies that promote transdisciplinary education can also add value to the designed interventions.^20^, ^21^, ^22^^,^^55^ For further research, to ensure proper design of such approaches, a crucial aspect that should be prioritized is the mapping of all AMR-related activities in the teaching programme. Despite following general frameworks and guidelines, dental curricula may show significant variations on the topic within countries and regions. The creation of dedicated interventions focussed on AMR and stewardship does not necessarily require the reshaping of the curricular structure and can promote awareness by engaging students enrolled in different years of study with joint assignments or campaigns. A good example are activities such as World Antimicrobial Awareness Week in November^56^ and the “Go Blue” campaign,^57^ which can yield positive results^58^ and provide periodic reinforcements to the teaching already provided.

Evaluating the impact of interventions in the same cohort of students would likely provide important data on progress over the years in terms of dental education and sustainability of the obtained results. This would also be valuable when evaluating characteristics inherent to each participant, such as involvement in research activities. The identification of cost-effective interventions aiming to increase awareness and promote education with the general public, young people, health care providers, and policymakers across world regions has been highlighted as one of the highest priorities in the World Health Organization global research agenda against AMR.^59^ In this study, the findings of higher awareness and higher confidence trends in the same direction were encouraging, as was the observation that involvement in research seems to engage students more in the perception of AMR as a global threat. The effectiveness of interventions and stewardship programmes should also be designed and evaluated locally in the Asia–Pacific region. As highlighted in a 2020 FDI white paper on the role of the dental team in reducing AMR,^13^ enhancing intercountry and interagency collaboration plays a central role in disseminating knowledge and technologies to fight AMR, as future prescribing generations are key stakeholders in addressing this global crisis.

## Conflict of interest

None disclosed.

## Appendix Supplementary materials

Image, application 1

## Footnotes

Supplementary material associated with this article can be found in the online version at doi:10.1016/j.identj.2024.09.016.
