# Supplementary material for: Insights Into Antimicrobial Resistance From Dental Students in the Asia–Pacific Region
Source: Int Dent J. 2024 Oct 5;75(1):263–72. doi: 10.1016/j.identj.2024.09.016 (PMC11806321; doi:10.1016/j.identj.2024.09.016)
Supplement: Supplementary file 1 [file mmc1.docx]

**Supplementary material**

|  | Vietnam | Sri Lanka | Japan | Australia  5-year program | Australia 4-year program | Total |
| --- | --- | --- | --- | --- | --- | --- |
| First year | 111 | 12 | - | 31 | 11 | 165 |
| Second year | 169 | 29 | - | 18 | 7 | 223 |
| Third year | 199 | 35 | 56 | 21 | 1 | 312 |
| Fourth year | 253 | 22 | 31 | 41 | 7 | 354 |
| Fifth year | 118 | 14 | 47 | 28 | - | 207 |
| Sixth year | 113 | - | 39 | - | - | 152 |
| Total | 963 | 112 | 173 | 139 | 26 | 1413 |

**Table S1.** Responses obtained by year in the different countries. Cells highlighted in green denote participants considered as final-year students (n=201).

***
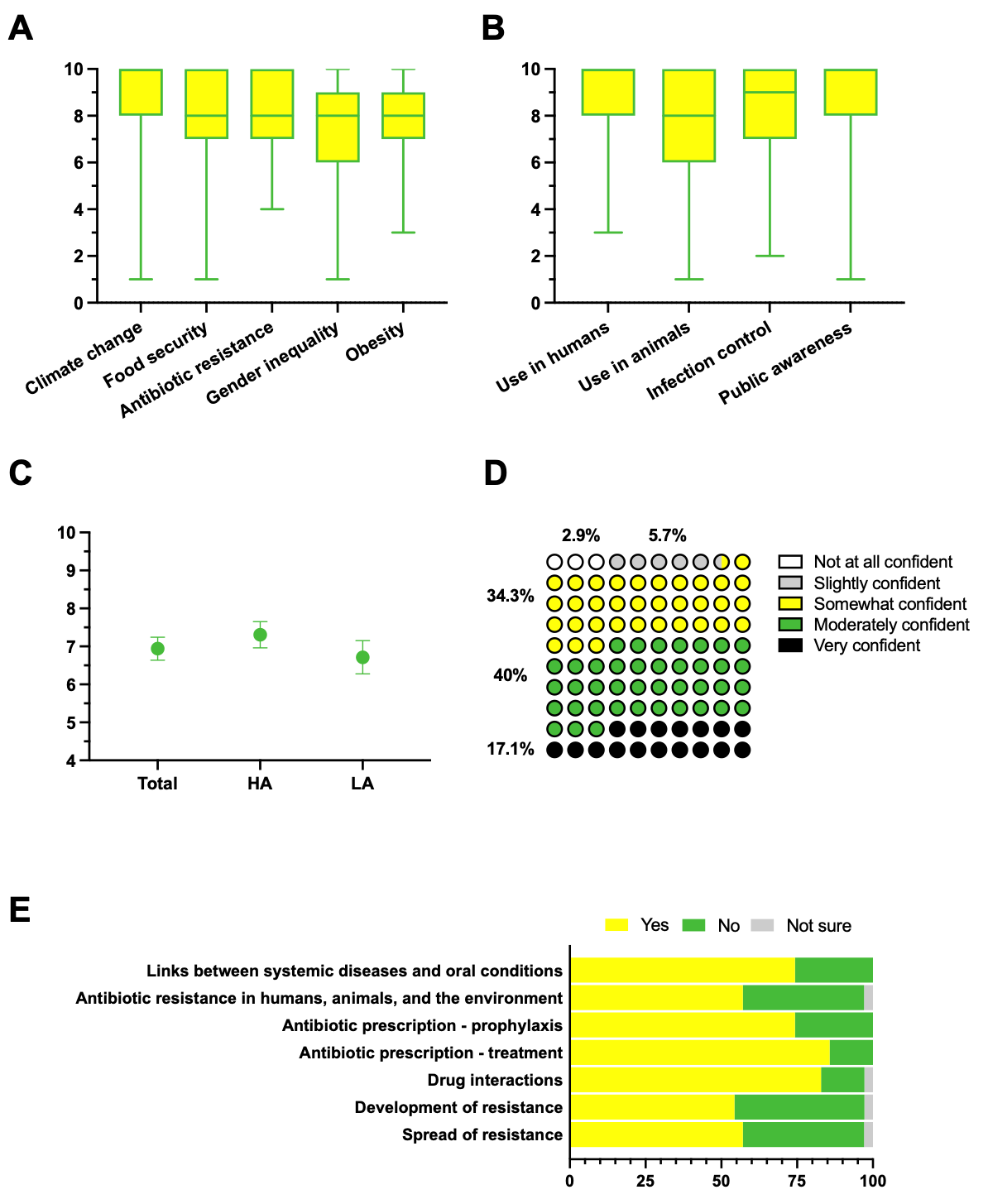
***

**Figure S1.** Data collected from participants in Australia. (A) Boxplot for perceived awareness on global issues, line indicates the median and whiskers indicate range. (B) Boxplot for areas should be addressed to slow down the development of antibiotic resistance. (C) Level of confidence of final year dental students to prescribe antibiotics on a scale from 1 to 10. Symbols show averages with error bars representing standard error of the mean (SEM). ‘T’ indicates total responses; ‘HA’—higher awareness on antibiotic resistance, meaning students that marked the challenge of AMR as either 9 or 10; ‘LA’—lower awareness on antibiotic resistance, meaning students that marked the challenge of AMR as 8 or below. (D) Level of confidence of final year students to tell patients when antibiotics are not needed. (E) Interest in receiving further education on different areas of microbiology and prescription practices.

***
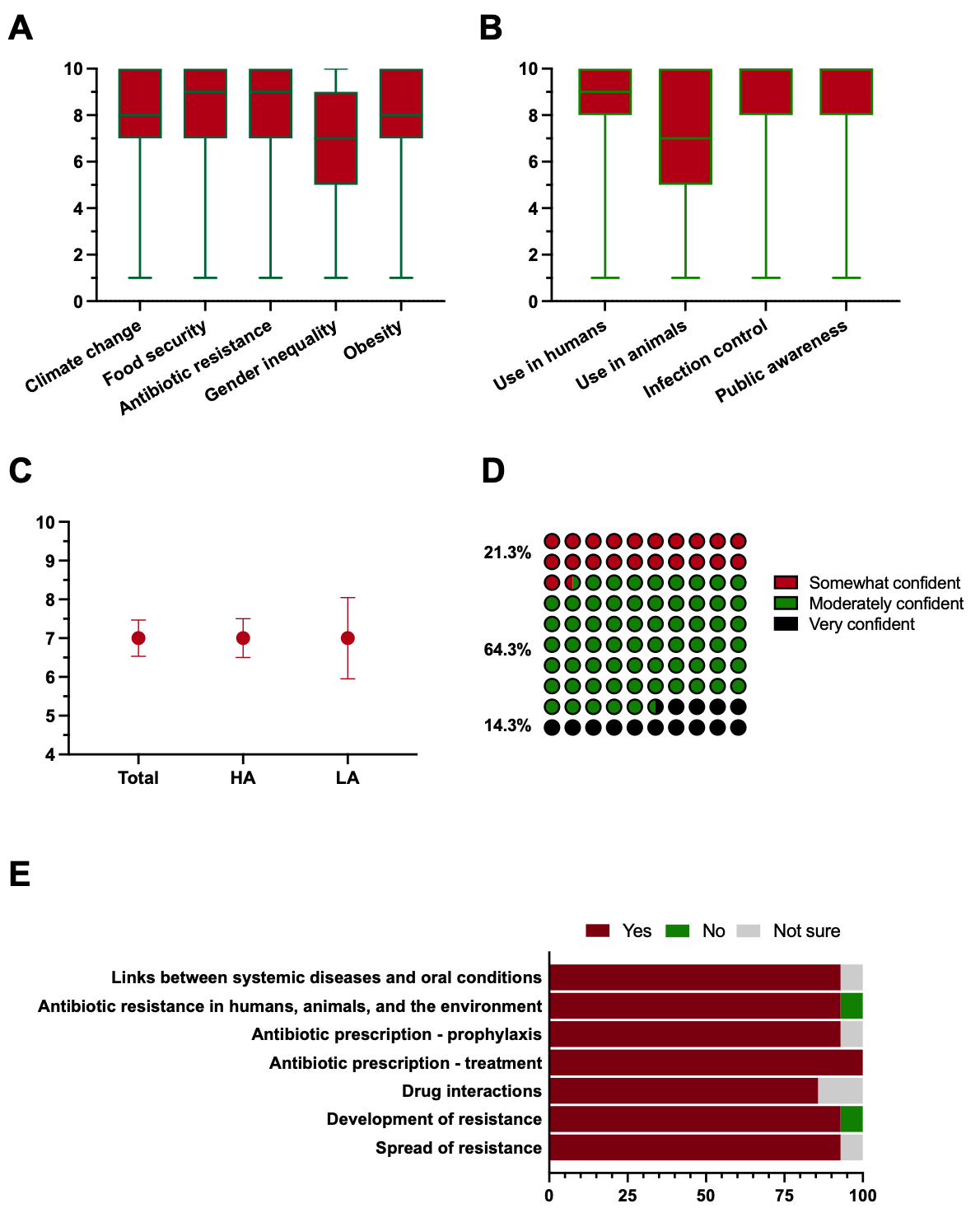
***

**Figure S2.** Data collected from participants in Sri Lanka. (A) Boxplot for perceived awareness on global issues, line indicates the median and whiskers indicate range. (B) Boxplot for areas should be addressed to slow down the development of antibiotic resistance. (C) Level of confidence of final year dental students to prescribe antibiotics on a scale from 1 to 10. Symbols show averages with error bars representing standard error of the mean (SEM). ‘T’ indicates total responses; ‘HA’—higher awareness on antibiotic resistance, meaning students that marked the challenge of AMR as either 9 or 10; ‘LA’—lower awareness on antibiotic resistance, meaning students that marked the challenge of AMR as 8 or below. (D) Level of confidence of final year students to tell patients when antibiotics are not needed. (E) Interest in receiving further education on different areas of microbiology and prescription practices.

***
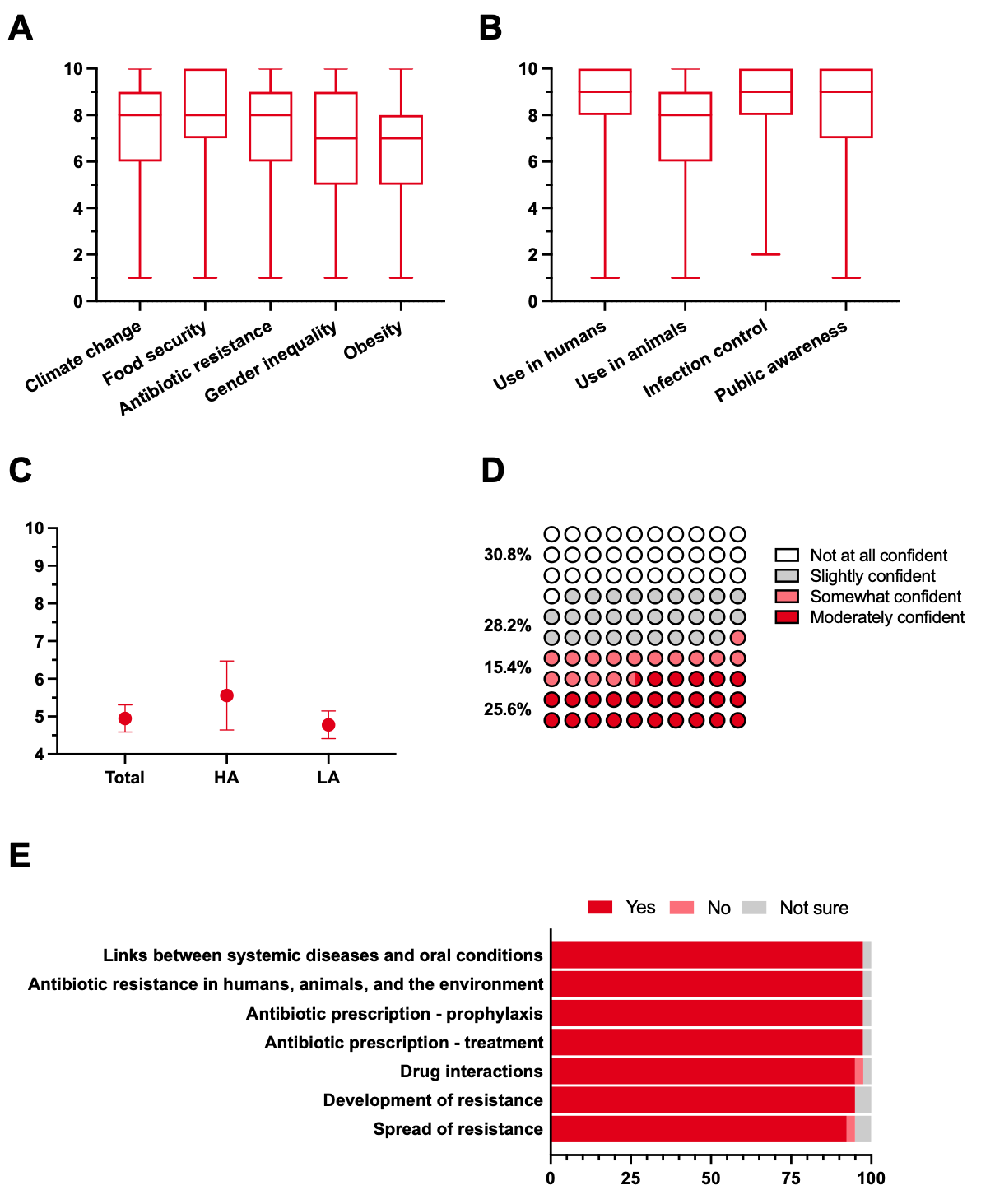
***

**Figure S3.** Data collected from participants in Japan. (A) Boxplot for perceived awareness on global issues, line indicates the median and whiskers indicate range. (B) Boxplot for areas should be addressed to slow down the development of antibiotic resistance. (C) Level of confidence of final year dental students to prescribe antibiotics on a scale from 1 to 10. Symbols show averages with error bars representing standard error of the mean (SEM). ‘T’ indicates total responses; ‘HA’—higher awareness on antibiotic resistance, meaning students that marked the challenge of AMR as either 9 or 10; ‘LA’—lower awareness on antibiotic resistance, meaning students that marked the challenge of AMR as 8 or below. (D) Level of confidence of final year students to tell patients when antibiotics are not needed. (E) Interest in receiving further education on different areas of microbiology and prescription practices.

***
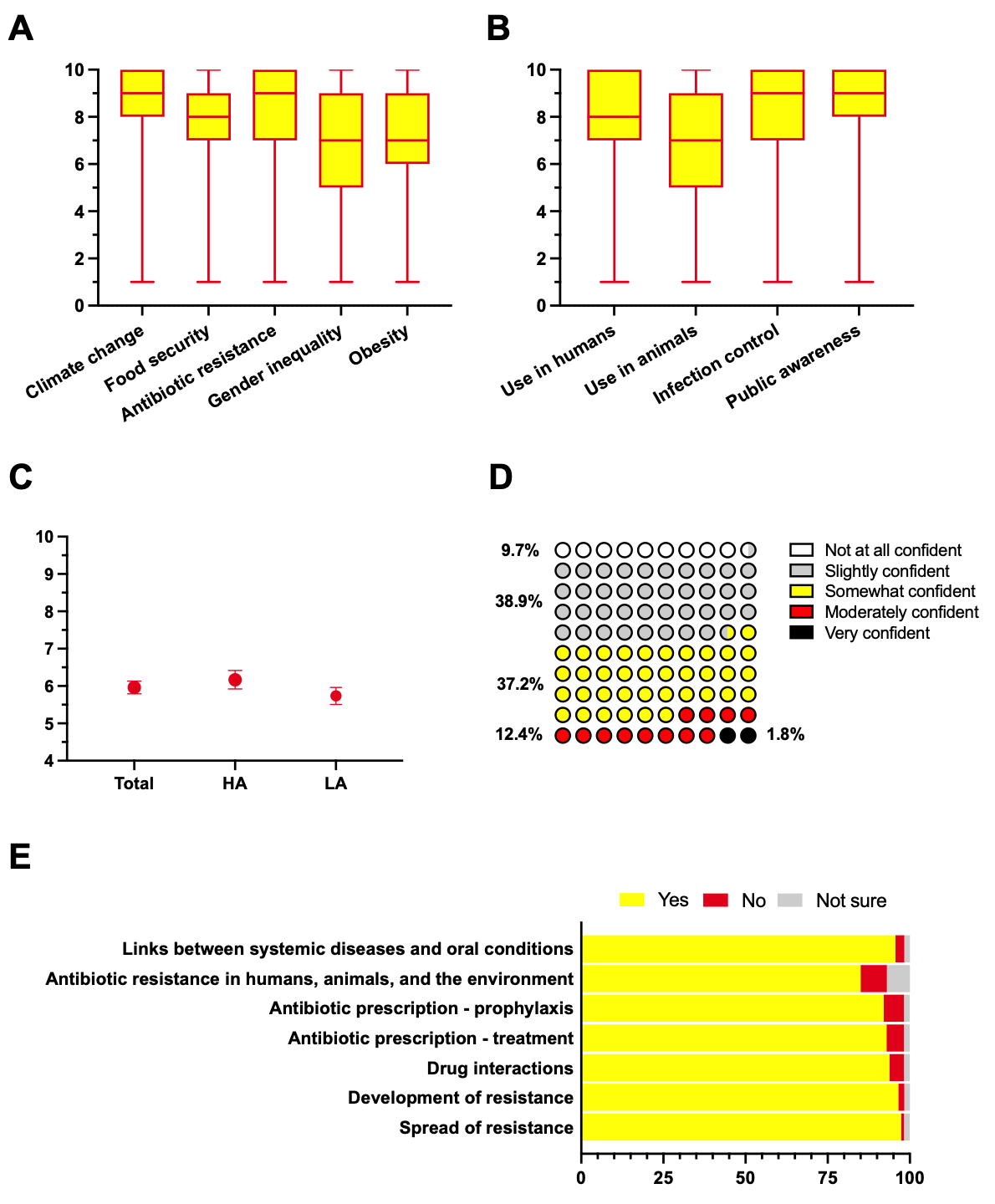
***

**Figure S4.** Data collected from participants in Vietnam. (A) Boxplot for perceived awareness on global issues, line indicates the median and whiskers indicate range. (B) Boxplot for areas should be addressed to slow down the development of antibiotic resistance. (C) Level of confidence of final year dental students to prescribe antibiotics on a scale from 1 to 10. Symbols show averages with error bars representing standard error of the mean (SEM). ‘T’ indicates total responses; ‘HA’—higher awareness on antibiotic resistance, meaning students that marked the challenge of AMR as either 9 or 10; ‘LA’—lower awareness on antibiotic resistance, meaning students that marked the challenge of AMR as 8 or below. (D) Level of confidence of final year students to tell patients when antibiotics are not needed. (E) Interest in receiving further education on different areas of microbiology and prescription practices.
